# The mediating role of healthy lifestyle behavior in the relationship between religious practice and academic achievement in university students

**DOI:** 10.1186/s40359-023-01455-1

**Published:** 2023-11-27

**Authors:** Kelemu Zelalem Berhanu, Abraham Abeje Shiferaw

**Affiliations:** 1 Department of Education Leadership and Management, Faculty of Education, University of Johannesburg, 524 Auckland Park, Johannesburg, South Africa Gauteng; 2 Department of Psychology, Debre Markos University, 269, Debre Markos, Amhara Region Ethiopia

**Keywords:** Religious practice, University students, Healthy lifestyle behavior, Academic achievement

## Abstract

**Background:**

The issues of religious practice, healthy lifestyle behavior and academic achievement are global agendas. Most previous research has focused on either one or two of the variables, not three of them (e.g. just the relationship between religious practice and healthy lifestyle behavior). And addressing these three issues, by and large, demands a systemic approach to re-thinking the current level and improving it.

**Objective:**

To examine the causal relationship between religious practice, healthy lifestyle behavior, and academic achievement in the case of Debre Markos University (DMU) and Injibara University (IU), Ethiopia.

**Methods:**

Four hundred forty students are participated voluntarily using random sampling techniques. To attain this objective, a cross-sectional research method design was used.

**Results:**

The mean scores of students’ healthy lifestyle behavior is more than average in all aspects. MANOVA results revealed that batch, ethnicity (region), and the university did not display a statistically significant difference among the composite (or combined) scores of both students’ healthy lifestyles and religious practice. However, religious affiliation and gender religious practice and have an effect on religious practice and healthy lifestyle behavior respectively. The correlation output informs that religious practice and healthy lifestyle behavior are positively and significantly correlated with each other. Religious practice also significantly predicted students’ healthy lifestyle behavior. Despite this, the academic achievement of students didn’t have any relationship with their religious practice and healthy lifestyle behavior.

**Conclusion:**

University students’ healthy lifestyle behavior doesn’t play an intervening variable in the effect of religious practice on academic achievement. Possible practical implications and recommendations have been forwarded.

## Introduction

A growing body of research recognizes the influences of religion and religious faith on human behavior and psychological functioning, particularly in adolescence [1]. Most university students are adolescence. Adolescence is a critical period of human development with rapid physical, psychosocial, cognitive, and emotional development, and sexual and reproductive maturation [2]. University students are vulnerable to unhealthy lifestyle behavior due to the nature of their academic life. Investing in university students’ health ensures triple dividends in terms of health during their adolescence, health during later adulthood as well as the health of the future generation [3].

### Religious practice

The first line of debate of the present study is about university students’ or adolescents’ religious practice. In this study, religious practice means a practice related to the holding of a religious belief. Dew et al. [4] found that the measurement of religious practice varied across studies in kinds of literature, with most studies defining religious practice as church attendance, religious beliefs, religious affiliation, or religious importance. For the purposes of this study, religiosity is defined as one’s beliefs and practices related to any religious affiliation or to God or Allah. A number of studies have found different factors or forms for religiosity: (1) public religiosity and (2) private religiosity. Private religiosity—is characterized by diverse, flexible, less restrictive social interaction and quiet faith, and less explicit religious activity and participation [5, 6]. For instance, it contains praying alone, and rarely participating in religious events and practices. Public religiosity which is characterized by membership in a clearly defined group, an individual’s centralized denominational social identification but relatively rigid. Public religiosity is the type of religious practice most commonly studied in the empirical literature [6]. Religious involvement may benefit adolescents’ lives by empowering both internal (e.g., the feeling of self-worth), social (e.g., a sense of belonging to a network) resources [7], and leading to ethical decisions [8]. Religious education can be instrumental in improving adolescents’ healthy lifestyle behavior by developing religious morality, reinforcing religious coping, developing respect for religious diversity, and promoting connectedness. Religious affiliation played a role in making difference in religious practice (Christianity has a high mean of religious practice, and Islam has the opposite [9].

### Healthy lifestyle behavior

A second line of debate in the present study is adolescents’ health lifestyle behavior. Healthy lifestyle behaviors are any determinants undertaken to prevent some kind of illness or to improve health and well-being [10]. The importance of the meaning of life in adolescent behavior is clearly reflected in human history [11]. Particularly, adolescence is a period when an individual develops the capacity to understand and internalize religion and its impact on their cognitive development. Adolescents are able to identify unhealthy lifestyles and healthy lifestyles. However, adolescents were engaged in healthy and unhealthy lifestyles simultaneously. This family, school, neighbors, and health care workers should work together and be vigilant in assessing and removing factors that prevent adolescents from adopting healthy lifestyles. Healthy lifestyle behavior include healthy eating, physical activity, sexual activity, and emotional well-being [12]; healthy physical and mental state (exercise, eating a healthy diet, and avoiding stress), vegetarian diet, no alcohol intake, an appropriate rest, increased recreational physical activity, development of their faith and hope, non-use of tobacco or other addictive substances.

Adolescents and young people are vulnerable to both a range of health risks and benefits [12]. Japar and Purwati [13] reported that the forms of adolescents’ unhealthy lifestyle behaviors are telling lies, going out without permission, staying up and talking all night, fighting with schoolmates, fighting with students, throwing trash anywhere, reading pornography books, watching pornography films, driving motorcycles/cars without driving licenses, driving at excessive speed, living together (out of matrimony), free sex, thieving, picking a pocket, committing armed robbery, abortion, rapping, killing. Health risks may affect adolescents immediately, such as infectious diseases, malnutrition, accidents, or sexually transmitted diseases, or in the future, such as cardiovascular diseases and cancers. These risks may originate as a result of their lifestyle and health status during adolescence [14].

Paweł et al. [10] identified four dimensions of lifestyle behaviors: Nutrition, prophylaxis (obeying health recommendations), positive attitude (avoiding emotional overload and stress situations), and pro-health practices (physical activity, good sleeping habits, and relaxation). However, there are currently other more popular approaches that consider multiple healthy lifestyle behavior. Thus, the present study uses a scale that measures university students’ healthy lifestyle behavior in various countries (e.g. in Saudi Arabia by Almutairi et al. [15]; in China by Wang et al. [16]; in Canada by Coulson et al. [17]. The dimensions of multiple healthy lifestyle behavior include: Healthy responsibility, nutrition, stress management, interpersonal relations, physical activity, and spiritual growth.

According to, Almutairi et al. [15], gender, type of college or university, and length of stay, or year in school were significant predictors of the healthy lifestyle of students in Saudi Arabia’s universities. Students in Saudi Arabia were found to have an inadequate level of adherence to recommendations regarding physical activity, and attend educational programs on health care and healthy nutrition habits. Coulson et al. [17] found that students had a lower level of stress, higher interpersonal relations, higher health responsibility, and better general spiritual health.

### Relationship between religious practice, healthy lifestyle behavior, and academic achievement

A third line of debate of the present study is about the relationship between religious practice, healthy lifestyle behavior, and academic achievement. When we examine the relationship between, religious practice and healthy lifestyle behavior, globally, a growing body of research recognizes religious involvement as an important dimension in adolescent development [1, 11].

Over the last decades, some researchers have studied the association between religiosity in health maintenance [18–23]. In addition, other studies have found that healthy lifestyle behavior is associated with religion and planned activities in Australia [18], Asia [24], American and Czech [25, 26] adolescents. It has been observed that adolescents and young people involved in some religious affiliation exhibit healthier behaviors, such as participating more actively in extracurricular activities during their free time [27–30] and getting more involved in family and religious social interaction [30, 31] than those who do not.

Gonçalves et al. [32] also showed that religious and spiritual interventions have positive effects on mental health outcomes such as a significant decrease in stress, depression, and alcoholism. Religion is affecting individuals’ choices with respect to risky behaviors by protecting individuals from risky behaviors. In Danish society, private religiosity facilitates social connection and healthy behavior to the same extent as more traditional social and participatory religiosity [6]. In particular, they found that religious individuals (publicly religious in focus) have healthier lifestyles compared to individuals with no religiosity. They also revealed the relationship between public religiosity and a healthy lifestyle—especially in terms of diet/ nutrition. It is true among Danish citizens too [33].

In the Danish community, in more religious cultures, there was a negative correlation between religiosity and health-related risk behaviors associated with a healthier lifestyle. On the contrary, healthier dietary patterns and less smoking were observed in people who had a strong tie to religious practice. Also path analysis and linear regression results, according to Paweł et al. [10] displayed that both spirituality and religiosity and health-related behaviors are positively related. Similarly, another study involving Muslim medical students in Iran showed that religiosity was protective against some unhealthy lifestyle behaviors and some psychological disorders such as depression [34]. This study conducted in Malaysia ascertained ethnicity and religion had adverse effects on health-related issues in general and psychiatric events in focus.

The Islamic religion in Malaysia, in particular, was found to be an important factor that protected the students from unhealthy lifestyle behavior including suicide attempts than Christianity, Hinduism, Buddhism, Taoism, and other religions [35]. However, another study among Jewish medical students found that there was no significant association between religiosity and healthy lifestyles behaviors such as depression, and anxiety [36]. In view of these mixed results, there is a need for more studies in this area to further define the relationship between religion practice and healthy lifestyle behavior. However, the results of related studies about the effect of religion are controversial.

Ample studies also revealed relationships between healthy lifestyle behavior and cognitive function in students [37–39]. Maniaci et al. [38] found that academic performance was negatively correlated with unhealthy lifestyle behaviors such as excessive internet use, perceived stress, and bad nutrition in Italian students. Composite scores of healthy lifestyles behavior were a significant predictor of better academic achievement.

More than 42 relevant studies supported that religion plays a causal role in the academic success of students [40]. For instance, in the Muslim dominant community, a study on students of the Islamic Religious Education Study Program disclosed that religiosity is positively and significantly related to student achievement motivation [41]. Similarly, a Christian-dominated population at a university in the United States revealed that religious affiliation and religiosity have an impact on students’ academic performance [9].

In Ethiopia, different studies were conducted in relation to the role of religious institutions in many affairs. For example, Kumilachew [42] has studied the role of churches in the provision of social support three types: emotional support, provision of food, and provision of cloths. In addition, he also reported that religion is inherently a social phenomenon and it has many effects on the socio-economic life of the community. Asselefech [43] has reported a remarkable finding on the role of Ethiopian churches in the development of adult education in Gonder and Addis Ababa. In addition to the aforementioned scholars, Serkalem [44] worked on the use, application, and integration of religious spirituality in clinical social service in Addis Ababa. In her study, she attempted to explore the contribution of religion in the process of helping patients in clinical social services. Tilahun et al. [45] also investigated the contribution of the Ethiopian Orthodox Tewahido church to forest management in the North Shewa Zone. Minychel [46] also explores the role of the Ethiopian Orthodox Tewahido Church (EOTC) contributes to mitigating the social problem.

The rationale for this research is based on four aspects. First, as we have observed above these studies in Ethiopia, previous researchers reflect or share some knowledge about the role of EOTC on natural resource conservation, mitigating social problems or conflict resolution, adult education, HIV/AIDS, and Health related issues. But, these studies conducted in Ethiopia, also show the same problem; which is focusing only on EOTC religion, which is similar to the previous one. All the above studies conducted in Ethiopia did not investigate about the role of religiosity and spirituality in shaping healthy lifestyle behavior and academic achievement. Second, the review of the previous studies demonstrated that mixed findings about the impact of religious practice. For example, numerous scholars such as Francis et al. [35] found that religious practice is important factor that enhance the students’ healthy lifestyle behavior. However, another study among Jewish medical students found that there was no significant association between religiosity and healthy lifestyles behavior [36]. There is a need for more studies in this area to further define the relationship between religious practice, healthy lifestyle behavior, and academic achievement. Thus, the foregoing scholars’ mixed findings about effects of religiosity motivated this investigation. Third, these three variables haven’t been studied jointly so far across globe. Fourth, this paper is believed to have significance for the officials of Ministry of Education (MoE), students, researchers, spiritual and secular organizations and other relevant stakeholders by increasing knowledge and information on the role religious practice in shaping adolescents’ healthy life style behavior and academic achievement. Based on the results of this study, educational policymakers at the federal level may include the development of religious capital as a means of promoting healthy lifestyle behavior. In addition, understanding healthy lifestyle behavior in adolescents is critical to the development of interventions needed to promote positive behaviors that can prevent negative physical and mental health outcomes, which may have lifelong implications. Thus, the findings of this study will be useful to the university adolescent students to be clear and ready for future life adjustment being religiously oriented and involved in their current life situation settings. Therefore, these initiations made researchers fill the gap by gearing towards the following basic research questions.

1. Is there a significant mean difference among university students in terms of their gender, religion, batch, region, and university regarding religious practice?
2. Is there a significant mean difference among university students in terms of their gender, religion, batch, region, and university regarding healthy lifestyle behavior?
3. Are there any significant relationship between students’ religious practice, healthy life style behavior, and academic achievement?
4. To what extent does students’ religious practice predict their healthy life style behaviors and academic achievement?
5. Does the healthy lifestyle behavior significantly mediate the relationship between religiosity/religious practice and academic achievement in University students?

### Theoretical framework

**Fig. 1:**
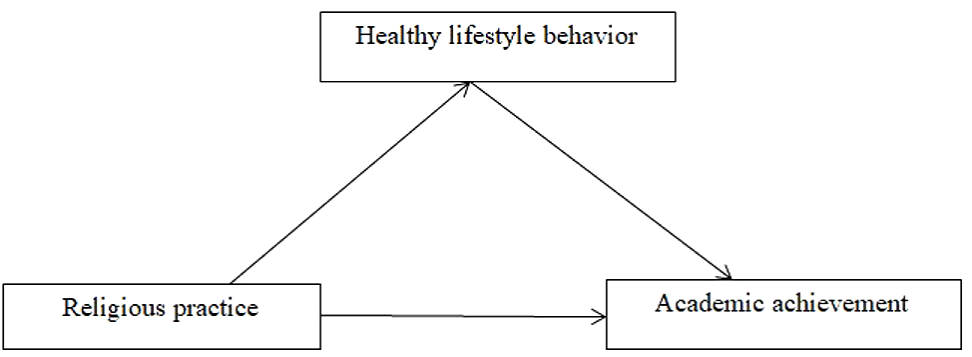
Proposed conceptual framework. (Figure 1 Alt Text: By using demands-resources (SD-R), researchers examine the role of various components of religious practice in healthy lifestyle behavior and academic achievement)

Researchers followed on study demands-resources (SD-R) model to test a model that examines the mediation effect of healthy lifestyle behavior in the relationship between religious practice and academic achievement. This theory is in line with job demands-resources model of Demerouti et al. [47] who stated that study resources (the good social or physical aspects at university) are associated with certain positive outcomes such as psychological and academic benefits. In the same vein, within this SD-R framework, high study resources foster positive outcomes, such as academic performance [48]. The major benefit of SD-R theory or model is that it is much more specific and focused on the university context. The SD-R framework also provides a theoretical basis to investigate the influences of the study context on students’ outcomes such as academic achievement, health and well-being. This model is an outstanding theoretical basis to examine the effects of the study context students’ success such as academic success, health, and well-being [48]. According to Lesener et al.’s [48] SD-R model, environmental resources promote study resources and produce positive study outcomes. This model is explicitly and exclusively validated within the university context. The present study, perceived religious practice (as one of the aspects of the environmental resources) may promote healthy lifestyle behavior (as one facet of study resources) and produce positive academic achievement as one of the study outcomes. The theoretical or conceptual framework is presented in Fig. 1.

## Methods

### Research design

The general objective of this study is to investigate the role of religious practice in shaping adolescents’ healthy lifestyle behavior and academic achievement. To attain this grand objective, the researchers employed cross-sectional research design. By using this systemic lens and aforementioned literatures, researchers can examine the role of various components of religious practice in healthy lifestyle behavior and academic achievement.

### Description of the area, population, sample size and sampling methods

The study was conducted at two public universities of Ethiopia: Debre markos University and Injibara University. These two universities are governed under the Ministry of Education (MoE), Federal Democratic Republic of Ethiopia (FDRE). For selecting universities and students, researchers used various sampling methods such as purposive sampling, proportionate stratified random sampling method and simple random sampling method. First, researchers started by deciding on the population of study by using purposive sampling method. Inclusion criteria for selecting population of the study were being governed under the Ministry of Education (MoE) or public universities, being applied or general universities and being located in East Gojjam province, West Gojjam province and Awi provinces of Ethiopia. These three provinces are thematic research areas of the funder of the present study (Debre Markos University). Private universities have been excluded from the study. Based on inclusion criteria, first, both Debre markos and Injibara universities are public universities; second, Debre markos and Injibara universities are classified as applied and general universities respectively; third, researchers conducted their study in the areas of three provinces of Ethiopia; East Gojjam province (Debre Markos university’s main campus), West Gojjam province (Debre Markos university’s Burie Campus), and Awi province (Injibara University).Therefore, undergraduate regular students who learn at Injibara and Debre Markos Universities served as sources of primary data. In addition, documents from the study site will be used as a secondary source of data. There are 6,940 (Male, M = 4,700; Female, F = 2,240) students in Debre Markos University (main campus-6228; M = 4246, F = 1982/ and Burie campuses; 712; M = 454, F = 258) in the academic year of 2021/2022. Injibara University has a total of 2,296 (1,173-M and Female-1,123) undergraduate regular students in the same academic 2013/14 Ethiopian Calendar, E.C (2021/2022 Gregorian calendar, G.C). Thus, this study has a target population of 9,236 (M = 5,873, F = 3,363).

Second, researchers decided total sample size. In this study, sampling sizes were determined using a simplified formula provided by Yamane [49]. That means:

n = N/1 + N (e) ^2^ = 9236/1 + 9236 (0.05)^2^ = 383 students (From Debre Markos University and Injibara University), Note: N = population size, n = sample size, *e =* level of precision. The most levels of significance used are 0.05 and 0.95 respectively. Since researchers may not know the standard deviation of the population they are studying, researchers should choose a number high enough to account for a variety of possibilities (such as 0.5).Third, furthermore, an additional 20% of the respondents were added to the recommended sample size [50] since the nature of the topic is potentially sensitive, the researchers expect that there would be non-response by some respondents either to a part of the questions or even to all of the questions. Accordingly, the total sample size was calculated to be (383+ (20/100 × 383)) = 460 students. Thus, the total number of participants was 460. Fourth, undergraduate regular students were selected from two universities by using a proportionate stratified random sampling method. That means based on the total population proportion, 114 students from Injibara and 346 students from Debre Markos University (Burie and main campus) were selected. In the last stage, from two universities, the researchers selected students using simple random sampling method.

### Data collecting methods

Questionnaires were used to assess the existing status of undergraduate students’ healthy lifestyle behavior, academic achievement, and religious practice. The questionnaire has four parts. 1) Personal information includes: (a) gender, (b) ethnicity/region, (c) religious affiliation (d) length of stay, (e) University, (f) CGPA (academic achievement); 2) students’ religious practice; 3) undergraduate students’ healthy lifestyle behavior.

This study employed two main instruments. In order to establish reliability and validity within this study, the following steps were implemented accordingly.

### Religious practice scale

Since religious practice in Ethiopia somehow different and context-specific, researchers decided to develop religious practice scale in the present study. In addition, Dew and colleagues [4] (2008) found that the measurement of religious practice varied across studies in kinds of literature. This is affirmed by Hinkin et al. [51] who stated an instrument development study might be necessary if a researcher determines that an existing instrument is inappropriate for use with their target population (e.g., cross-cultural issues). The development of a scale for religious practice was conducted based on the scale development recommendations of Lamm et al. [52]. First, researchers begin by reviewing a list of indicators found in very useful publications related to religious practice [52]. Thus, for this study, the religious practice scale for university students was compiled from different scholars [53, 54]. For more detailed or deeper information, researchers gathered data through interviews with religious leaders of Islam and Christianity. Before administrating, the questionnaire was given to two academics who were experts on the topic to ensure its content validity [52, 55]. Initially, 24 items were developed upon interview responses and a previously developed questionnaire. Then a questionnaire was distributed to 440 university students. When internal consistency analyses were conducted using Cronbach’s alpha coefficient to obtain correlated item-total correlation, three items (R3, R10, R20) didn’t have a correlation of moderately high to high (e.g., 0.40+). These items were deleted since alpha increases if these Items (items 3, 10, 20) were deleted [56]. Thus 21 items were left after conducting internal consistency analyses. In order to determine the pattern or dimensions of the university students’ religious practice scale, exploratory factor analysis was performed. The data on factor load values and the total variance of the items are given in Table 1.

**Table 1:** Exploratory factor analysis of religious practice scale

Items	Factor load values	
	Factor 1	Factor 2
I am fasting based on the religious’ liturgies	0.729	
I am doing religious deeds based on the religious’ liturgies, canon law, dogmas	0.724	
I am in a state of readiness to do spiritual activities based on the religious’ liturgies, canon law, and dogmas	0.657	
By having an advice from Father Confessors or religious leaders), I get solutions for my own current problems	0.648	
I practice all religious’ liturgies and deeds	0.623	
I listen religious education from religious leaders	0.620	
The frequency where I am going to holy places (churches, Mosques, etc.)	0.615	
Since I am a religious person, I do good things	0.591	
I have a religious leader (Father Confessor, Sheik, Pastor, etc.) for the purpose of counseling service	0.588	
I respect all religious’ liturgies	0.536	
I pray to supernatural force	0.604	
As I am a religious person, I like assisting persons living within poor situations; like feeding for hungry, drinking for thirsty, wearing for non-dressing, etc.		0.570
Since I am a religious person, I strive to make persons more beneficial wherever		0.555
Our supernatural force helps me by sending His Angels Missionaries) while I am becoming in a state of challenges		0.776
Supernatural force has His own time to do for His deeds		0.743
Since I am a religious or Laity man, I am living and working peacefully with human beings		0.717
Since human beings are well respected by supernatural force, and then I give a respect for males and females equally		0.710
I believe by the deed of supernatural force faithfully		0.624
% of Variance	36.168	11.103
Cronbach Alpha	0.86	0.75

Total Variance Explained rate = % 47.27

Total Cronbach Alpha = 0.89

Kaiser-Meyer-Olkin Measure of Sampling Adequacy = 0.883

Bartlett’s Test of Sphericity Approx. Chi-Square(X^2^ (153) = 13298.15; *p* < 0.01))

Extraction Method: Principal Component Analysis.

Rotation Method: Varimax with Kaiser Normalization.^a^

N.B. Factor 1- Faith-based personal practice and factor 2- Religious Social Support/Activities

In this study, as shown in Table 1, the KMO value was 0.883; the Bartlett Sphericity test was (X2 (153) = 13298.15; *p* < 0.01) Factor analysis yielded a two-factor solution that explained 47.27% of the variance. According to Akgül and Çevik [57], KMO higher than 0.60 showed that was meaningful and adequacy for factor analysis. It was concluded that the sample size was very good enough to make a factor analysis. In factor analysis, three items (items R5, R6, R11) were not up to the requirement of factor loading and were deleted from the analysis. Finally, findings obtained from exploratory factor analysis showed the university students’ religious practice scale has two factors or dimensions with 18 items. Even if names of sub-dimensions is completely subjective and up to researchers, researchers tried to use more appropriate names by examining previous studies. Meaningful names for the extracted sub-dimensions should be given [51]. Researchers took previously used factor names (faith-based self-practice and religious social support or activities) by various scholars due to consistency of items [4–6]. Internal consistency analyses were conducted using Cronbach’s alpha coefficient to obtain reliable estimates for both factors, and a composite score. Cronbach’s alpha coefficient demonstrated strong internal consistency estimates (α = 0.86 for Faith-based personal practice (11 items e.g. I am fasting based on the religious’ liturgies), α = 0.75 for Religious Social Support/Activities (7 items e.g. since I am a religious man, I am living and working peacefully with human beings), α = 0.89 for the composite (18 items)). Field [58] stated that if the alpha coefficient is between 0.60 and 0.80, the scale is moderate and fair, between 0.80 and 1.0; the scale is a higher level of reliability. In this respect, it can be said that the reliability of university students’ religious practice scale is from moderate to high.

### Healthy lifestyle behavior

In this study, through adaptation, a reliable and valid scale that measures university students’ healthy lifestyle behavior was used. It was implemented in Saudi Arabia by Almutairi et al. [15]; in China by Teng et al. [59] and Wang et al. [16]; in Canada by Coulson et al. [17]. They have quite a similar number of items (around 40) and dimensions. Sample items were: I comply with health professionals’ advice and health guidelines; and I make an effort to monitor my emotional changes. In this study, construct validity was supported by exploratory factor analysis, which yielded a six-factor instrument that explained 52.34% of the variance in the 29 items. These are Healthy responsibility, nutrition, stress management, interpersonal relations, physical activity, and spiritual growth. The factors were in line with some of the aforementioned scholars [17]. Eleven items were deleted due to overlapping with various factors and low factor loading. In order to know the fitness of the instrument for this study, a confirmatory factor analysis using LISREL8.7 program was performed and confirmed with undergraduate regular students who were randomly selected from 2 universities in Ethiopia. The *P*-value is the first value to be examined in confirmatory factor analysis and is significant at the 0.00 level. T values from parameter estimates exceed 1.96, and it is significant at the 0.05 level [60]. The goodness of fit indices of healthy lifestyle behavior scale is presented in Table 2.

**Table 2:** Goodness of fit indices of healthy lifestyle behavior scale

Fit indexes	Acceptable Boundary /Criteria	Goodness of Fit Values for the Study
X2/SD	≤ 2 = Perfect fit	X2/SD = 3.38
	≤ 2.5 = Perfect fit (small population)	
	≤ 3 = Perfect fit(large population)	
	≤ 5 = Moderate fit	
GFI/AGFI/ NFI/NNFI/IFI/RFI/CFI	≥ 0,95 = Perfect fit	GFI = 0.9, AGFI = 0.81, NFI = 0.89 NNFI = 0.91, IFI = 0.92, RFI = 0.93, CFI = 0.95
	≥ 0,90 = good fit	
RMSEA/RMR/SRMR/	≤ 0,05 = Perfect fit	RMSEA = 0.07, RMR = 0.055 SRMR = 0.041
	≤ 0,08 = Good fit	
	≤ 0,10 = weak fit	

The instrument had acceptable level of goodness of fit since IFI, RFI, NNFI and CFI indices were over 0.90 [60]. Cronbach’s alpha coefficient for composite scores was 0.859.Healthy lifestyle behavior Scale for University Students has good construct validity and reliability and can be used in this study and in university health centers.

### Data analyses techniques

The data collected using a questionnaire was analyzed through quantitative analysis techniques (both descriptive and inferential statistics). For instance, descriptive statistics (frequency count, percentage, and mean) were used to analyze demographic data and the extent to which students’ healthy lifestyle behavior, and students’ religious practice-. In this study, inferential statistics such as MANOVA were utilized in order to assess the influence of demographic factors such as gender, religion, region (ethnic), batch, and university as independent variables and students’ healthy lifestyle behavior, and religious practice as dependent variables [61]. As a standard level of significance, α = 0.05 has been taken as many scholars suggested [62]. To measure effect sizes, partial eta squared (ƞ2) was employed. Regression analysis was also utilized to measure the influence of student’s religious practice on their healthy lifestyle behavior. Path Analysis with Lisrel 8.7 was employed to see the direct and indirect effects of religious practice on students’ academic achievement mediated through healthy lifestyle behavior.

## Results

### Return rate

Out of 460 distributed questionnaires, 440 (95.65%) were returned. That means 20 questionnaires were removed because of the incompleteness of data. Questionnaire response rates are very adequate because as a rule, a response rate as low as 50% is possible for surveys [61] (Cohen et al., 2018). The participants of this study incorporated students from various personal characteristics such as gender, religion, region, and batch/length of stay in university. It was also collected from two public universities proportionally to a total number of students in each university.

#### Religious practice of university’ students

As displayed in Table 3, the mean scores of students’ religious practice are more than average in both aspects. In addition, the correlation relations among the DVs of students’ religious practice revealed that there is a moderate and positive correlation among each composite variable. If the correlations were above 0.60, we would take the sum /average or eliminate one of the variables [56]. The correlation output implies that a successful practice in one component of religious practice complements the same output in the other. This smoothed the way for running MANOVA to examine whether there are significant mean differences among the socio-demographic variables.

**Table 3:** The bivariate correlation among religious practice of university’ students

Dependent variables	Mean	Std. Deviation	Faith-based self-practice	Religious social support/activities
Faith-based private practice	43.4718	8.15545		0.638^**^
Religious social support or activities	30.9545	4.08706		

Note: **Correlation is significant at the 0.01 level

*Correlation is significant at the 0.05 level

As displayed in Table 4, the effect for religion was significant, Wilk’s A = 962, F (6,722) = 2.325, *p* < 0.05, η2 = 0.019, and η = 0.137. This reveals that the linear composite of students’ religious practice differs for orthodox, Islam, protestant, and other religions. Follow up post hoc test was conducted for the religion to find out exactly where the mean score differences are since just religion revealed significant differences. Mean differences have been observed in composite sets of students’ religious practice between orthodox and other (Mean of Orthodox = 74.36, Mean of Other = 65.05, *p* < 0.05);. Similarly, there were differences in the same variable between Islam and other (Mean of Islam = 77, Mean of Other = 65.05, *p* < 0.05) and protestant and other (Mean of protestant = 76.3, Mean of Other = 65.05, *p* < 0.05). It can be reported other religions such as catholic, seven Adventist, and traditional believers tone down religious practice when compared with orthodox, Islam, and protestant students. In particular, the post hoc test also revealed a nearly similar trend with respect to students’ faith-based personal practice, too. However, with respect to religious social activities or support, mean differences have not been observed.

**Table 4:** Multivariate analysis of religious practice of students

Effect	Wilks’ Lambda Value	F	Hypothesis df	Error df	Sig.	Partial Eta Squared (η2)	Eta (η)
Gender	0.999	.151^b^	2.000	361.000	0.860	0.001	0.031
Religion	0.962	2.325^b^	6.000	722.000	0.031	0.019	0.137
Batch	0.992	.754^b^	4.000	722.000	0.556	0.004	0.063
Region	0.972	1.726^b^	6.000	722.000	0.112	0.014	0.118
University Y	0.992	1.542^b^	2.000	361.000	0.215	0.008	0.089

However, gender, batch (length of stay in university), region, and the university did not display a statistically significant difference among the composite (or combined) scores of students’ religious practice. (Wilk’s Λ = 0.999, F (2, 361) = 0.151, *p* > 0.01, η = 0.031), (Wilk’s Λ = 0.992, F (4, 722) = 0.754, *p* > 0.05, η = 0.063), (Wilk’s Λ = 0.972, F (6, 722) = 1.726, *p* > 0.05, η = 0.118) and (Wilk’s Λ = 0.992, F (2, 361) = 1.542, *p* > 0.05, η = 0.089) respectively. Here effect size test or η2 or η value is very small. This implies that in the current study gender, batch, region, and university played no role in students’ composite sets of religious practice. In other words, this MANOVA result suggests that the actual difference in the mean values is a very small and linear composite of religious practice are not differ in terms of gender, batch, region, and university.

#### Healthy lifestyles of university’ students

Table 5 has shown, the mean scores of students’ healthy lifestyle are more than average in all aspects. Also all correlations among each composite variable are in between low and moderate. There were no variables which have correlations of above 0.60. A correlation of above 0.60 runs the risk of multicollinearity [56]. The correlation output indicated that a successful practice in one component of healthy lifestyle complements the same output on the other. In between low and moderate correlation among composite sets of DVS paved the way for running MANOVA to examine whether there are significant mean differences among the demographic variables such as gender, religion, batch, region and university.

**Table 5:** The bivariate correlation among healthy life styles of university’ students

DVs	Mean		Healthy responsibility	Spiritual growth	Interpersonal relations	Stress management	Nutrition	Physical activity
Healthy responsibility		6.7932		0.300^**^	0.210^**^	0.186^**^	0.281^**^	0.188^**^
Spiritual growth		26.2159			0.521^**^	0.450^**^	0.271^**^	0.283^**^
Interpersonal relations		24.1273				0.347^**^	0.144^**^	0.156^**^
Stress management		13.2818					0.345^**^	0.322^**^
Nutrition		14.4841						0.596^**^
Physical activity		17.7864						

Note: **Correlation is significant at the 0.01 level

*Correlation is significant at the 0.05 level

As indicated in Table 6, the effect for gender was significant, Wilk’s A = 0.962, F (6,357) = 2.526, *p* < 0.05, η2 = 0.041, and η = 0.202. This reveals that the linear composite of students’ healthy lifestyle behavior differs for males and females. Follow-up ANOVA indicates that an effect of gender was significant for both physical activities and composite scores of healthy lifestyle behavior. Males scored higher on physical activities (mean of male = 18.84, mean of females 16.19, *P* < 0.01). Mean differences have been also observed in composite sets of students’ healthy lifestyle behavior between males and females (Mean of Male = 101.48, Mean of female = 97.96, *p* < 0.05). It can be seen that female students tone down practicing healthy lifestyle behavior when compared with males. However, with respect to healthy responsibility, interpersonal relations, stress management, spiritual growth, and nutrition, just a little means differences have been observed.

**Table 6:** Multivariate analysis of healthy lifestyle behavior of students

Effect	Wilks’ Lambda	F	Hypothesis df	Error df	Sig.	Partial Eta Squared	Eta (η)
Gender	0.959	2.526^b^	6.000	357.000	0.021	0.041	0.202
Religion	0.958	0.853	18.000	1010.234	0.638	0.014	0.118
Batch	0.987	.396^b^	12.000	714.000	0.965	0.007	0.083
Region	0.926	1.550	18.000	1010.234	0.066	0.025	0.158
University	0.986	.834^b^	6.000	357.000	0.544	0.014	0.118

However, MANOVA result revealed that religion, batch, region, and the university did not display a statistically significant difference among the composite (or combined) scores of students’ healthy lifestyle behavior (Wilk’s Λ = 0.958, F (18, 1010.234) = 0.853, *p* > 0.05, η2 = 0.014), (Wilk’s Λ = 0.987, F (12, 714) = 0.396, *p* > 0.05, η2 = 0.007), (Wilk’s Λ = 0.926 F (18, 1010.234) = 1.55, *p* > 0.05, η2 = 0.025) and (Wilk’s Λ = 0.986, F (6, 357) = 834, *p* > 0.05, η2 = 0.014) respectively.

#### Relationship among students’ religious practice, healthy life style behavior and academic achievement

In this part of the study, the relationship between religious practice, healthy life style behavior, and academic achievement is presented.

Table 7 depicted the relationship between religious practice, academic achievement, and healthy lifestyle behavior. The correlation output informs that the two variable groups (religious practice and healthy lifestyle behavior) are positively and significantly correlated to each other except for physical activities and religious social support or activities. That means, when students are good in their religious practice so do they in their healthy lifestyle behavior. In this respect, it can be said that an increase or a successful practice of religion will lead to an increase in any one of the healthy lifestyle behavior of students except for a few factors (between physical activities and religious social support or activities). However, the academic achievement (CGPA) of students didn’t have any relationship with their composite score of religious practice (r=-0.086) and composite score of healthy lifestyle behavior (r = 0.096).

**Table 7:** Correlation analysis of university students’ religion practice, healthy lifestyle behavior and academic achievement

	Health respons.	Spiritual growth	Interpersonal relations	Stress manag.	Nutrition	Physical Activity	Composite healthy life style behavior	Academic achievement
Faith-based self-practice	0.149^**^	0.361^**^	0.337^**^	0.243^**^	0.240**	0.173^**^	0.386**	− 0.113^*^
Religious social support or activities	0.103^*^	0.393^**^	0.402^**^	0.191^**^	0.101*	0.089	0.33**	− 0.011
Composite score of religious practice	0.725**	^*^0.406**^**^	0.396^**^	0.246^**^	0.21^**^	0.158^**^	0.401**	− 0.086
Academic achievement	− 0.066	− 0.024	− 0.009	− 0.085	− 0.110*	− 0.045	0.096	1

Note: **Correlation is significant at the 0.01 level

*Correlation is significant at the 0.05 level

#### The effect of students’ religious practice on their healthy lifestyle behavior and academic achievement

It is a good idea to check the correlations among variables prior to running the linear regression, to determine if either the variables are sufficiently correlated such that multicollinearity is highly likely to be a problem or no relationship [56]. Here in this study depicted there is no relationship between composite score of religious practice and academic achievement, thus researchers omitted it due to violation of the assumption of regression of analysis. However, linear regression analyses are predicted healthy lifestyle behavior from students’ religious practice. The adjusted R2 indicates that we have a fairly good model, explaining about 15.9% of the variance in healthy lifestyle behavior (R = 0.401, R^2^ = 0.161, Adjusted R^2^ = 0.159, ΔR^2^ = 0.161, *P* < 0.01). According to Cohen [63], this is a good effect. The composite scores of religious practice significantly predicted students’ healthy lifestyle behavior (F (1,438) = 83.885, *P* < 0.01). When the t-test result related to the significance of regression coefficients was examined, it is seen that religious practice was an important predictor of healthy lifestyle behavior.

#### Mediating role of healthy lifestyle behavior on the relation between religious practice and academic achievement in university students

**Fig. 2:**
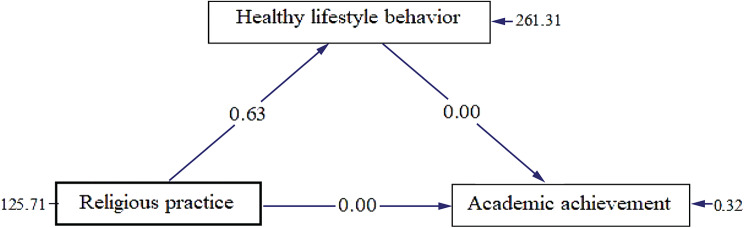
Path analysis results of the mediating role of healthy lifestyle behavior on the relationship between religious practice and academic achievement

Path Analysis with Lisrel 8.7’s result of the direct and indirect effect of religious practice on students’ academic achievement mediated through healthy lifestyle behavior was presented below in Fig. 2.

According to the results of Fig. 2, the path results showed students’ healthy lifestyle behavior has no significant effect on their academic achievement (B = 0.0), Healthy lifestyle behavior doesn’t play a mediating variable on the association between religious practice and academic achievement because the total value is not greater than the direct effect. The path analysis result from Lisrel 8.7 also revealed that the indirect effect of religious practice is 0 (0.63*0.0). Thus, it appears that the indirect and direct effect of total religious practice on academic achievement (0) is not greater than the direct influence (0.0). These results indicate that students’ healthy lifestyle behavior doesn’t play as an intervening variable in the effect of religious practice on academic achievement.

## Discussion

To piece empirical studies on mediating role of healthy lifestyle behavior on the relationship between religious practice and academic achievement, this paper used Study Demands-Resources (SD-R) model. This model offers a comprehensive view on, demands, resources, and outcomes, and on the interplay of different antecedents. This study investigated the extent to which the strength (in terms of extent) of students’ healthy behavior mediates the association between composite scores of religious practice (religious social activities and faith-based private practice) and academic achievement. Our results displayed that the mean scores of students’ religious practice is more than average in both aspects. These depicted that the students accomplished what is expected of them both religiosity factors. Hence a successful practice in one component of religious practice complements the same output in the other. A comparison of mean scores on religious practice among students in terms of demographic variables didn’t demonstrate the existence of significant differences, except with respect to religion. Other religious affiliations such as catholic, seven Adventist, and traditional believers are tone down religious practice when compared with orthodox, Islam, and protestant students. In line with the present findings, religious affiliation played a role in making difference in religiosity [9]. However, the current final output contradicts with earlier findings of Li and Murphy [9] who found that Christianity has a high mean of religiosity, and Islam has the opposite.

These findings, like the religious practice, notify that students under investigation are good enough in their healthy lifestyle behavior. In a similar vein, Coulsonet al. [17] found that students in Canada had a lower level of stress, higher interpersonal relations, higher health responsibility, and better general spiritual health. The finding didn’t comply with those of Almutairi et al. [15] who reported that students in Saudi Arabia have an inadequate level of adherence to recommendations regarding physical activity, and attend educational programs on health care and healthy nutrition habits. The correlation output also indicated that a successful practice in one component of healthy lifestyle behavior complements the same output on the other. MANOVA output indicated the effect for gender was significant. This reveals that males scored higher on physical activities and composite sets of students’ healthy lifestyle behavior. The results from the previous study like the results from the present study found there was gender difference [15].

In the current findings, with respect to healthy responsibility, interpersonal relations, stress management, spiritual growth, and nutrition, just a little means differences have been observed. However other MANOVA results of the present study revealed that religion, batch, region, and the university did not display a statistically significant difference among the composite (or combined) scores of students’ healthy lifestyle behavior. On contrary with the present findings, Almutairi et al. [15] found that the type of college or university, and batch (length of stay) were significant predictors of the healthy lifestyle of students in Saudi Arabia’s universities. Inconsistently to the present result, in Malaysia, ethnicity and religion had adverse effects on health-related issues in general. The Islamic religion in particular was found to be an important factor that protected the students from unhealthy lifestyle behavior than Christianity, Hinduism, Buddhism, Taoism, and other religions [35].

According to students’ actual practice and behaviors, correlation coefficients among the different religious practice and healthy lifestyle behavior confirmed positive relationships – some with low to moderate except for physical activities and religious social support or activities. This implies that a successful religious practice complements the same output on healthy lifestyle behavior. This aligns with the outputs by Sulaiman [8] who found that religious education can be instrumental in improving adolescents’ healthy lifestyle behavior by developing reinforcing religious coping, developing respect for religious diversity, and promoting connectedness. Our results align with many past studies. For instance, a previous study found a positive association between public religiosity and a healthy lifestyle in a sample of Danes [33].

When we link with past study findings conducted in many countries (such as in Australia [18] Asia [24], American and Czech [25, 26]) that claimed adolescents involved in religious activities exhibit healthier behaviors. In addition, it has been observed that adolescents involved in some religious affiliation are participating more physically activity in extracurricular activities than those who do not [29, 30].

Francis et al. [35], Gonçalves et al. [32], and Heidari, et al. [34] also showed that religious interventions have positive effects on mental health outcomes by protecting against some unhealthy lifestyle behavior and some psychological disorders such as depression. Similarly to the present findings, recently, Paweł et al. [10] and Svensson [33] revealed the relationship between public religiosity and healthy lifestyle—especially in terms of diet/ nutrition. This finding contradicts other earlier findings who unveiled that there was no significant association between religiosity and healthy lifestyles behaviors among Jewish medical students [36]. Therefore, it is important for school to create a caring and socially supportive environment for religious practice, and conduct extra-curricular activities to keep students engaged in healthy lifestyle behavior.

The correlation coefficient depicted that the academic achievement of students didn’t have any relationship with their composite scores of religious practice and healthy lifestyle behavior. This finding contradicts other earlier findings such as Burns et al. [37], Maniaci et al. [38], Owen et al. [39] who found there was a relationship between healthy lifestyle behavior and cognitive function in students. More than 42 reviews of relevant studies, inconsistent with the current findings, also supported that religion plays a causal role in the academic success of students [40]. For instance, in the Muslim dominant community, a study on students of the Islamic Religious Education Study Program disclosed that religiosity is positively and significantly related to student achievement motivation that affects academic achievement directly [41]. Christian-dominated populations at a university in the United States also revealed that religiosity has an impact on students’ academic performance [9].

According to the path results of current findings, students’ healthy lifestyle behavior has no significant effect on their academic achievement (B = 0.0), Inconsistency with the present findings, Maniaci et al. [38] found that composite scores of healthy lifestyles behaviors were a significant predictor in better academic achievement. The present findings also depicted that healthy lifestyle behavior doesn’t play a mediating variable in the association between religious practice and academic achievement because the total value is not greater than the direct effect. A possible reason that no healthy lifestyle behavior mediation was detected in the present study might be to do with the fact that there was very little variance in the healthy lifestyle behavior sum score between the religiosity and academic achievement. Finally, to our knowledge, this is the first study to investigate whether healthy lifestyle behavior mediates the association between religious practice and academic achievement among university students.

### Limitations

This study was limited by the study sample, university students. In the future, it can be expanded by including secondary or high school students to illuminate how healthy lifestyle behavior can be influenced by religious practice, how religious practice and healthy lifestyle behavior may influence their academic achievement, and how behaviors of healthy lifestyles, academic achievement, and religious practice can be promoted. The findings of the present study are also restricted to the concepts chosen, which may preclude other variables from being discovered. For example, social networks, and life satisfaction by Lim and Putnam [7] and psychological well-being by Paweł et al. [10] are a mediator between spirituality or religiosity, and health-related behavior but these variables were not included in the present study due to restrictions in survey length. Thus, future studies from adding other elements that include a mediator or moderator variable, such as family demographic characteristics, leadership, students’ engagement, motivation and can be investigated.

Furthermore, methodologically this study was delimited to quantitative research. Because cross-sectional, a quantitative methodology was used, it was not possible entirely to discover disputes that may have negatively impacted academic achievement, healthy lifestyle behavior, and religiosity such as parent-related cultural, educational, and socioeconomic factors. Similarly, qualitative data-gathering instruments such as interviews with religious leaders may highlight new ideas about the relationship between religious practice and healthy lifestyle behavior. Hence, this study can be investigated both by using qualitative and quantitative research methods. Furthermore, data were collected once at a point at which is very difficult to see the causal relationship between variables. If the issue is given an excessive amount of time to study by using the longitudinal methodology, it is possible to see the actual causal relationship between academic achievement, healthy lifestyle behavior, and religious practice.

## Conclusion and implications for research and practice

Ultimately, by distinguishing various factors of religious practice and healthy lifestyle behavior, this study represents an initial step towards uncovering the nature of the association among different variations of religious practice, healthy lifestyle behavior, and academic achievement. Our results indicated healthy lifestyle behavior has far-reaching consequences and is associated with religiosity over non-religiosity. It is plausible to come up with a kind of conclusion that in the current study area, religiosity has no far-reaching consequences on academic achievement. Recently, in the 21st century due to secularism, modern university study programs focused on fulfilling the material needs of individuals, however often lack deep philosophical content about the paramount importance of religiosity, which should play a significant role in shaping the meaning of life, healthy lifestyle behavior and reflecting on the purpose of life. It is assets investing in the development of resources associated with spirituality. Therefore, the results of the current study may find some practical application in the area of education to focus on religious education.

In this study, plausibly, various remedial mechanisms are commendable. Even if the sampled universities are somehow good at implementing health promotion programs, the management of the universities should plan and implement programs to motivate students to be much more responsible for their own health in general. Thus, it can be concluded that developing and implementing goal-oriented programs to promote dimensions of healthy lifestyle behavior such as stress management, health responsibility, interpersonal relations, physical activity, spiritual growth, and nutrition may promote healthy lifestyles among university students.

The results of our study can also guide universities and educators in promoting information initiatives about the importance of religious practice as a means to not only reduce unhealthy lifestyle behavior but also achieve life goals since religiosity has played a role in shaping healthy lifestyle behavior, as our findings. Educators and health-related professionals in focus should also pay more consideration to the link between religious practice and healthy lifestyle behavior.

## Data Availability

The datasets used and/or analysed during the current study available from the corresponding author on reasonable request.
